# Notes on the Detection of Hydrocyanic Acid, in Medico-Legal Cases

**Published:** 1870-01

**Authors:** M. J. DeRosset

**Affiliations:** Professor of Chemistry, Balto. College of Dental Surgery.


### ARTICLE III.

### *Notes on the Detection of Hydrocyanic Acid, in Medico-Legal Cases,*

By M. J. DeRosset, M. D.,

Professor of Chemistry, Balto. College of Dental Surgery.

It is a fact worthy of note, that in many of the "causes celebres," the history of which is preserved in the annals of forensic medicine, where the question of the guilt hinges upon the chemical evidence, attempts have been made to invalidate the testimony of the analyst, often upon the most insubstantial pretext. The plan usually adopted by the ingenious counsel is not to attack the general method of the analysis, but by studying carefully the evidence, and by measuring every step, to seize upon any omission, however trivial, as the key note to the tone of his argument.

The neglect of some minor element in the process is most often the chief factor in lessening the force of the testimony although an over-abundance of manipulation is occasionally instrumental in introducing weak links into the chain. These, therefore, omission and redundancy are the Syclla and Charybdis through which the chemist must pass, and it is important to look well to the possible dangers on either side, if he would keep within the limits of safety, and obtain results so clear that none may withstand the force of their logic.

A strict regard for the honor of the science, no less than for the individual credit, should cause the same careful conservatism to be carried into the witness box that has been employed in the research. And as it is essential to avoid hasty deductions in the laboratory, so it is well in testifying to permit no extraneous matter, brought to light during the trial, to mould or alter one form or figure of the conclusions to which the results of the analysis, alone considered, have led.

It is of the highest import to know whether the poison found be a constant or occasional constituent of the body, and whether it may be formed as a result of post-mortem changes. These are questions to which the chemist may rightly be called on to give definite replies, and his information in this direction should be of the most positive character. His office, in a word, is to instruct the judge and jury as to matters of fact, but beyond this "*Vir sapit qui pauca loquitur.*" Speculations as to the relations between the chemical facts and the remaining testimony form no part of the chemist's duty; the calculation of these probabilities belongs to others, and he is wise who declines to be led away by the counsel from the clear summary which his experiments have developed.

If the usual tests give no evidence of poison, it is the duty of the chemist to testify that none was present, although the strongest chain of evidence seems to point to one. When it is a question of fixed poisons, as arsenic or the alkaloids, which do not readily undergo change, then a failure to elicit the characteristic reactions is a certain evidence of the non-presence of them; but if discovered they are always to be connected with the ante-mortem circumstances, since there is no possibility that any one of them shall be generated by the living or dead body, or from its substance by any process of analysis.

The case differs somewhat for gaseous and volatile poisons of organic origin, as hydrocyanic acid, cyanogen, and even the poisonous cyanides. If the nature of the poison be unknown, these volatile ones should be first sought after, the simplest methods being preferred, at first tentatively, and afterwards for full corroboration.

It must not be forgotton that *cyanogen compounds are not infrequently found among the products of the natural decomposition of animal tissues ; further, the identical composition of urea and cyanate of ammonia is to be remembered ; and that the destructive distillation of uric acid gives rise to cyanic and hydrocyanic acids.* These are possible sources of traces of the poison, and together with the *sulpho-cyanogen of the saliva* are to be considered *quoad* their value in the final conclusions. In view of these facts the deductions must be drawn with the greatest care. If but faint traces are obtained it must be so stated, and, in conjunction therewith, all the possible conditions under which such traces might be exhibited should be carefully and fully detailed, but whatever connection any one of them may bear to the general evidence must be left to be declared by the court.

With the chemist it is solely a question of the presence or absence of poison, and whether or not death ensued therefrom, but *under no circumstances is he justified* in forming and expressing a belief in the guilt or innocence of the accused.

The great volatility of the poison renders it important that the analysis be made speedily, and once uudertaken, it must be conducted through to its completion without interruption. If putrefaction has commenced, any hydrodcyanic acid present will be in the form of a cyanide or sulphocyanide of ammonium, which may be dissolved out by alcohol, the mixture if alkaline or neutral being acidified with tartaric acid, and the whole distilled at a low temperature (178° F.). The tests for the vapor are to be applied to the distillate; and here it may be stated that it is not within the power of the analyst to discriminate between hydrocyanic acid and any cyanide, the reactions in each being due only to the cyanogen.

Let us examine briefly the various tests and the fallacies into which they may lead us.

*The nitrate of silver* test is very available, exceedingly delicate in its results, but the white cyanide of silver which forms, if cyanogen is present, may be confounded with the white chloride of the same metal which would result from the presence of hydrochloric acid. The distinction between these two salts is sufficiently characteristic, with appreciable quantities, but the delicacy of the test will also exhibit such minute quantities of either as to render it impracticable to distinguish between the two. Nevertheless the test should always be used, at least as an exploratory one; if it gives traces of a white silver salt, although too small to be further dealt with, hydrocyanic acid may be suspected, and proven by Scheele's and Liebig's methods. If sufficient traces are obtained there is the further inestimable advantage of the characteristic cyanogen flame which may be had from the cyanide of silver.

Schoenbein has lately suggested a new method for detecting minute traces of hydrocyanic acid. It is useful for exploratory purposes but is of no value whatever in fixing a crime. It is as follows:

Unsized (filtering paper) paper is soaked in a tincture of guaiacum, containing 3 per cent. of the resin, and dried. When to be used it is moistened with a solution of sulphate of copper, of the strength of one or two grains to the ounce, when if brough into contact with the vapor of hydrocyanic acid instantly it is rendered blue, It is said that one drop of the acid in 4000 ounces of water may be detected by this test. But in addition to its being valueless in medico-legal cases, it is open to the still further objection of exhibiting a similar blue color in ammonia vapor, from the formation of ammonio sulphate of copper. The main corroborating tests are Scheele's and Liebig's.

*Scheele's test.*—Caustic potash and hydrocyanic acid in presence of each other unite to form the white cyanide of potassium; a mixture of a ferrous and a ferric salt added to this yields a permanent blue precipitate, the ferrocyanide of iron (Prussian blue).

But a bluish precipitate is also the result of mixing these salts of iron with caustic potash when *no* hydrocyanic acid is present, the hydrated ferric oxide being formed, and the test would be inconclusive if arrested at this stage; so hydrochloric acid is added in slight excess which causes the ferric oxide to dissolve, but the Prussian blue remains, if any was present, and must be taken as a proof of the presence of hy drocyanic acid.

The chances of error are two fold.

First the Prussian blue does not always form immediately if the quantity of the poison be small, but requires often an hour or two for its appearance; and hence we may conclude too hastily that no poison is present, when a longer lapse of time would have demonstrated it.

Secondly, The precipitated ferric oxide may be mistaken for Prussian blue, especially if any remain after an insufficient addition of hydrochloric acid, and thus we may believe that we have detected the poison even when none was present.

Liebig's test is exceedingly delicate and conclusive, and offers but two chances of error, and these remote ones.

First, if the sulphydrate of ammonium is not evaporated to dryness, a black sulphide of iron is apt to form on the addition of a ferric salt, and thus mask the red sulpo-cyanide of iron.

Again the sulpho-cyanide of iron may be confounded with the acetate or the meconate of iron, both of these salts being red; so the test should always terminate with the addition of corrosive sublimate which bleaches the sulpho-cyanide, but does not effect either the acetate or meconate of iron. It is proper to add that neither of these latter substances are apt to be present, yet the possibility calls for the proper discriminating test.

A few words in conclusion respecting the chances for the detection of this poison after death. If death has been sudden from a large dose it may be detected for a notable period thereafter ; in one instance where two or three drachms had been swallowed traces of it were obtained three weeks after burial. But this is unusual. If death was gradual, it will certainly not be readily detected, and especially we may not expect to find it in the *stomach more easily than elsewhere, for, being so diffusible, it would speedily disappear from that viscus during life.*

It is, therefore, in such cases quite as important to select other portions of the body for analysis as that organ, and if there be choice of any it will most probably, if the conjecture may be hazarded, lie in the great nerve centres upon which the whole violence of this subtle poison is expended, destroying life as if by a blow, not simply, we may say, by creating some mysterious vital impress there, but, in a more intelligible way, by satisfying its powerful affinities from among the physiological elements of nerve structure.

